# An Integrated Review of Findings from the Early Head Start University Partnerships Buffering Toxic Stress Consortium

**DOI:** 10.1007/s11121-025-01801-2

**Published:** 2025-04-26

**Authors:** Neda Senehi, Aleta Meyer, Mary Bruce Webb, Sangeeta Parikshak, Alysia Blandon

**Affiliations:** 1 Office of Planning, Research and Evaluation, Administration for Children and Families, US Department of Health and Human Services, 330 C Street SW, Washington, DC 20201 USA; 2 Office of Head Start, Administration for Children and Families, US Department of Health and Human Services, 330 C Street SW, Washington, DC 20201 USA

**Keywords:** Toxic stress response, Parenting enhancements, Early Head Start, Buffering, Precision home-visiting

## Abstract

This paper introduces the special issue of *Prevention Science* titled *Applied Prevention Science to Inform Parenting Enhancements to Early Head Start that Promote Supportive Parent–Child Interactions and Buffer the Detrimental Effects of Early Adversity.* We provide an integrative overview of the Early Head Start-University Partnerships Buffering Toxic Stress (BTS) consortium, funded by the Administration for Children and Families (ACF), summarizing key findings from six studies and reflecting on their implications in light of recent ACF and Office of Head Start (OHS) policies and regulations. Programs such as Early Head Start (EHS), which integrate parenting support and skill-building, have demonstrated effectiveness in enhancing parental competencies, reducing stress, and improving child outcomes. However, intervention effects remain modest and vary significantly by factors such as race and ethnicity, demographic risk, and family characteristics. This variability underscores the need for tailored, culturally responsive approaches that validate and refine frameworks for understanding parental and social buffering of children’s toxic stress response. Findings from the BTS studies highlight promising program impacts, especially for families facing heightened risk, such as those with maternal histories of adverse childhood experiences or ongoing mental health challenges. This synthesis reinforces the importance of precision-based, contextually responsive interventions and aligns with recent ACF and OHS regulations aimed at developing scalable, cost-effective models that meet the unique needs of families experiencing adversity. Beyond EHS, we hope this special issue advances the field of applied prevention science by informing the development of tailored, effective, and sustainable programming for children and families navigating early adversity.

Parenting interventions hold significant promise in buffering children from the negative impacts of unmitigated adversity, often referred to as a toxic stress response, by fostering responsive caregiving, enhancing parent–child interactions, and promoting secure attachments (Masten & Barnes, 2018; Shonkoff et al., 2012). This special issue addresses the growing need for research on optimizing preventive parenting interventions to meet the unique challenges faced by diverse Early Head Start (EHS) eligible families who often experience multi-level, multi-factor, and elevated adversity. Programs such as EHS, which integrate parenting support and skill-building, have demonstrated effectiveness in enhancing parental competencies, reducing stress, and improving child outcomes, particularly in families experiencing multiple and chronic adversity (Harden et al., 2012; Love et al., 2005). Parenting programs have also been effective in reducing young children’s problem behaviors (Leijten et al., 2019; Mingebach et al., 2018) and prevention of child maltreatment (Chen & Chan, 2016). However, home-based and center-based preventive parenting interventions’ effectiveness remains small and significantly varies based on subgroup differences in race and ethnicity, demographic risk, family characteristics, and risk and protective factors, highlighting the need for tailored interventions for EHS families (Raikes et al., 2013; Supplee & Duggan, 2019) and validation of frameworks for understanding the construct of parental or social buffering of a toxic stress response in diverse EHS populations with young infants and toddlers from birth to two years (Hostinar et al., 2014).

Despite the clear need and benefits, increasing the reach of tailored and intensive parenting interventions remains challenging (Supplee & Metz, 2015). The necessity for greater precision in adapting interventions to individual family needs often results in increased cost, reducing scalability and accessibility (Fagan et al., 2019). This limitation further compromises implementation, access, and uptake of evidence-based parenting interventions, particularly for minoritized families experiencing heightened risk (Bornstein et al., 2022; Harden et al., 2012). Additionally, a significant gap remains in understanding how systemic adversity challenges evidence-based parenting program implementation, uptake, and effectiveness (Fagan et al., 2019). In fact, recent overviews of evidence focusing on home visiting that are relevant to EHS emphasize the importance of advancing a *precision approach* to achieve greater effectiveness and ensure successful, tailored implementation for families by better understanding what works best for whom (Home Visiting Applied Research Collaborative [HARC], n.d.; Supplee & Duggan, 2019). Thus, there is a significant need for research focused on developing and validating contextually responsive frameworks to comprehensively identify risk and protective factors that impact the tailored implementation and evaluation of preventive parenting interventions, to ultimately improve child and family well-being for all families.

The six papers presented in this special issue use the toxic stress framework (Shonkoff et al., 2009) to characterize and examine parental buffering and toxic stress responses from birth to age two. The papers offer insights on how parenting interventions can be adapted to mitigate the harmful effects of prolonged toxic stress on EHS children and their families. The goal of the current integrative overview is to provide background on the EHS-University Partnerships Buffering Toxic Stress (BTS) consortium funded by the Administration for Children and families (ACF), summarize and synthesize findings across the six papers from the BTS consortium, and to reflect on recent ACF and Office of Head Start (OHS) regulations and policies considering these findings. In turn, the aim of the special issue is to offer a body of evidence that informs the development of tailored, evidence-based preventive parenting enhancements that promote resilience and disrupt the intergenerational transmission of adversity in diverse EHS populations. Beyond EHS, we hope this special issue contributes to the development of tailored, effective, and sustainable programming for children and families facing early adversity.

## Toxic Stress Framework

A pivotal advancement in this area of parenting interventions emerged in 2009 when Shonkoff and colleagues synthesized research on the links between early adversity and lifelong physical and mental health outcomes and introduced the framework of toxic stress. Toxic stress refers to the frequent, strong, and prolonged stress response activation that is experienced without the protection of a supportive caregiving relationship (Shonkoff et al., 2009). This framework emphasizes the critical role of sensitive and emotionally supportive adult–child interactions in buffering children from experiencing a toxic stress response (Shonkoff et al., 2009). Based on this psychobiological developmental framework, a compelling hypothesis emerged that parenting interventions can effectively strengthen parent–child relationships, which in turn *buffer* infants and toddlers from the harmful effects of environmental stressors, thereby disrupting the intergenerational transmission of adversity (Shonkoff et al., 2012).

Consistent with the toxic stress framework, research has identified parental support and positive parent–child interactions as key mediational pathways through which EHS programming exert lasting effects on young children’s developmental outcomes in EHS families (Raikes et al., 2014). However, not all EHS families equally benefit or sustain program gains. For instance, while Raikes et al. (2013) found that African American families showed the greatest sustained gains in maternal supportiveness, no significant effects were observed for their White or Hispanic families. These findings underscore the need to hone and rigorously validate the toxic stress framework to better understand mechanisms (e.g., active ingredients) that drive program impacts, particularly for populations that are harder to serve, such as Latinx^1^ families, who represent more than half (53%) of the participants across all studies in this special issue (with a range of 11 to 82%; Phu et al., 2021). This underscores the need for tailored early preventive interventions to promote health and prevent disease in EHS families.

This special issue responds to a growing need to more precisely capture the construct of toxic stress and to evaluate the added effectiveness of parenting enhancements within EHS families living in the context of adversity. The manuscripts included in this special issue seek to validate and expand upon the toxic stress framework, presenting innovative empirical research that examines various dimensions of toxic stress. These studies range from testing theoretical foundations, exploring etiological and contextual factors, validate measurement constructs, and explore moderation effects in efficacy studies. The six studies presented in the special issue were conducted within everyday early childhood program settings and facilitated through collaborative research-practice partnerships between university researchers and EHS grant recipients.^2^ This body of applied developmental research makes a significant contribution to translation and adoption of evidence-based knowledge about early adversity into prevention practices. These findings also demonstrate the critical role EHS can play in effectively promoting parenting practices that buffer children from experiencing a toxic stress response and disrupting the intergenerational transmission of adversity.

## Evidence on the Role of Supportive Parenting in Buffering the Impacts of Early Stress Across Development

There is compelling evidence that childhood experiences of toxic stress response are associated with elevated risk for poor mental and physical health outcomes across the lifespan. This exposure can also compromise caregivers’ capacity to provide supportive parenting, perpetuating an intergenerational cycle of adversity (McEwen & McEwen, 2017; Shonkoff et al., 2012). Furthermore, early childhood represents a particularly sensitive period due to heightened brain plasticity, which makes young children more susceptible to both positive and negative experience-dependent and experience-expectant outcomes (Knudsen, 2004). For instance, children who experience economic hardship during childhood show greater cognitive deficits in adulthood, in part due to compromised stress regulation (Evans & Schamberg, 2009). On the other hand, children who receive greater maternal support during early childhood show significantly larger hippocampal volumes, which are associated with better memory and stress regulation during school age, even after controlling for socioeconomic status and exposure to stressful life events (Luby et al., 2012). These findings exemplify a well-established body of evidence that emphasizes the protective role of supportive parenting in mitigating the long-term effects of early stress through putative biological and behavioral pathways in rodent, non-human, and human primates (Gunnar et al., 2015; Obradović & Boyce, 2009).

## The Need for the Validation of Toxic Stress Framework

One goal of the special issue papers is to examine the validity of the toxic stress framework when applied and translated into practice. While the toxic stress framework offers valuable insights into how early adversity impacts development, there remain gaps in translating developmental science into effective intervention outcomes. Evidence from research using the toxic stress framework suggests that parenting interventions can buffer children from harmful effects of early stress on health and development (Shonkoff et al., 2009, 2012). These interventions focus on fostering secure parent–child relationships and emotionally responsive caregiving that promotes resilience among children at risk for experiencing toxic stress response (unmitigated). Additionally, they underscore the importance of addressing caregivers’ capacity across systems of influence and their experiences of adversity (Shonkoff & Fisher, 2013).

Despite its theoretical promise, significant challenges exist in translating and applying these findings into the development of effective and scalable interventions. Interventions designed to replicate what has been observed in developmental research do not always produce the anticipated changes in children’s developmental trajectories (Blair & Raver, 2012). This gap may result from inconsistencies in translating evidence into practice, as well as the challenges of implementing theoretical frameworks within the constraints of real-world intervention settings. Variability in contextual factors, such as family and child characteristics, and the fidelity with which interventions are adopted and executed, further complicate the translation of developmental theory into practice. Rather than being a limitation, context is an essential and often overlooked factor that significantly influences long-term success of interventions. Therefore, while developmental frameworks are valuable for designing interventions, their replicability, scalability, and impact must be rigorously evaluated within specific subgroups and real-world contexts (Raikes et al., 2013; Supplee et al., 2018). A shared goal of the special issue papers is to illustrate and validate the proposed pathways and constructs that are central in the toxic stress framework through rigorous evaluation in EHS settings with varied populations (Buffering Toxic Stress Consortium Principal Investigators et al., 2013).

## The Role of Precision to Buffer Toxic Stress: What Works for Whom Under What Conditions

The toxic stress framework highlights the need to consider child, family, and broader contextual factors to reduce the impact of stressors on child outcomes (McEwen & McEwen, 2017; Shonkoff et al., 2012). Addressing both adversity and resilience through an early childhood psychobiological lens further underscores the importance of tailored, preventive parenting interventions that produce sustained impacts for all families (Supplee & Duggan, 2019). One promising approach in improving efficiency and effectiveness of parenting interventions has emerged as “precision home-visiting,” which involves adapting home-based interventions to account for multiple levels of influence, including individual, family, program, and community-specific contextual factors. This approach emphasizes identification of active ingredients (“what works”) that drive impact across meaningful subgroups (“for whom”; HARC, n.d.; Haroz et al., 2019; Supplee et al., 2018).

Precision home-visiting is a promising approach to enhance effectiveness and scalability of preventive early childhood interventions (Supplee et al., 2018), and its lessons are highly relevant to EHS settings and populations. This aligns well with research grounded in the toxic stress framework, which highlights significant variability in stress reactivity across individuals and contexts. Consistently, research has demonstrated that EHS program effectiveness on parenting and child outcomes vary as a function of demographic risk and parental well-being including parental stress and psychopathology (Brophy-Herb et al., 2022; Love et al., 2005). Additionally, a well-established body of research highlights the reciprocal relationship between parenting practices and parental well-being across diverse contexts (Nomaguchi & Milkie, 2020). Moreover, variability in temperament, parent–child interactions, and family stress profiles further underscores the need for tailored approaches to accommodate these children's and families’ unique characteristics (Masten & Cicchetti, 2016). Given this evidence, it is reasonable to hypothesize that tailoring interventions to these unique characteristics enhances their “buffering” effectiveness. Therefore, precision in intervention design is a necessary next step in the field to increase efficiency and effectiveness particularly for families experiencing high levels of adversity.

Furthermore, validating the toxic stress framework and informing a precision approach requires that researchers examine contextual factors that moderate intervention effectiveness. This is especially critical for families with infants and toddlers between 0 and 3, a period that is uniquely sensitive to the development of brain and biological systems underlying stress regulation (Lupien et al., 2009) and long-term health outcomes (Obradović & Boyce, 2009). Parenting interventions during this critical developmental window have the potential to influence long-term health outcomes, making it essential to adopt a “precision approach” to home-based interventions (Supplee & Duggan, 2019). Such approaches will ensure that interventions are tailored to the specific needs of diverse families, improving both uptake and long-term impact on child and family wellbeing.

## Early Head Start: an Evidence-Based Program and Learning Laboratory for Comprehensive Child Development and Family Strengthening Services

Early Head Start (Office of Head Start, n.d.), an evidence-based home visiting program for disadvantaged infants, toddlers, and pregnant women, provides comprehensive two-generation child development and family strengthening services. Similar to its sister program, Head Start, EHS has followed the tradition of serving as a “national laboratory” for child development research. Since its inception in the mid-1990s, EHS partnered with the research community to design and implement child development practice, evaluate program outcomes, and guide continuous program improvement. This collaborative research-practice partnership approach has not only advanced the field of child development research but has also provided real-world applications that have moved the field forward in research and in how EHS programs are implemented.

A prominent example of these research-practice partnerships is the Early Head Start Research and Evaluation Project, a multisite, multi-informant, randomized evaluation conducted by a consortium of researchers and EHS program partners across 17 sites (Love et al., 2002). This large-scale evaluation, which followed children until the fifth grade, has generated extensive evidence on the risks and protective factors among parents and caregivers within the EHS population related to program, child, and family outcomes (Love et al., 2013).

Despite the well-documented benefits of EHS services, access to these programs remains disproportionately low among families facing multiple adversities (Baxter et al., 2022). In fact, only about 10% of eligible children are enrolled in EHS programs (National Head Start Association, 2023), with barriers including limited program slots, high teacher turnover rates, long waitlists, transportation issues, lack of awareness about eligibility, and insufficient service hours further compounding the problem (National Head Start Association, 2023; Sandstrom et al., 2024). Additionally, the co-occurring adversities faced by EHS families may compromise participant engagement, a crucial factor in the success of parenting programs (Forry et al., 2011). For instance, the main EHS impact evaluation study (Love et al., 2002) revealed that in addition to demographic risks such as unemployment, welfare receipt, single or teen parenthood, and low educational attainment and about half of the parents reported high levels of depression. These findings highlight the complex challenges faced by EHS programs who must identify and support children and parents at increased risk. Additionally, EHS program are required to promote both parenting and family self-sufficiency, as federally mandated by the high standards for comprehensive two-generation services set by the Head Start Performance Standards (Office of Head Start, 2024a).

These systemic barriers highlight the urgent need for targeted outreach efforts and policies that address access to EHS, particularly for subgroups experiencing elevated adversity (Schmit & Walker, 2016). The challenge of disproportionate access to EHS is increasingly relevant to the focus of this special issue, which advocates for the importance of adopting a precision paradigm in early childhood interventions. As the research presented in this special issue demonstrates, the variability in families’ needs—such as those stemming from high levels of caregiver psychopathology, adverse childhood experiences, economic hardship and resource constraints, and child difficult temperament- calls for interventions that are not only evidence-based but also tailored to the specific risk profiles of different family subgroups to maximize effectiveness. This approach is exemplified by EHS’s role as a “national laboratory” for child development research where programmatic refinements and targeted enhancements, such as parenting interventions, can be developed and tested in real-world settings.

EHS’s success in supporting the developmental needs of infants and toddlers is validated by its inclusion on the Home Visiting Evidence of Effectiveness (HomVEE) list of evidence-based practices for the Maternal, Infant, and Early Childhood Home Visiting (MIECHV) program (Sama-Miller et al., 2018). However, a significant gap in the HomVEE evidence-based programming is the limited evidence on how effective home-visiting models are for different types of families with diverse characteristics (Sama-Miller et al., 2018). While studies often include diverse samples in terms of race/ethnicity and socioeconomic status, sample sizes are usually too small to analyze results by subgroup (Sama-Miller et al., 2018). However, to fully realize the potential of EHS within the precision paradigm, there is a pressing need to ensure that the most vulnerable families—those with multiple and co-occurring adversity—are reached and can benefit from these interventions. This special issue contributes to this goal by advancing our understanding of how precision-focused frameworks can help buffer toxic stress and improve outcomes for children and families facing the highest levels of adversity.

## A Consortium of Early Head Start—University Partnerships to Inform Precision in Buffering Toxic Stress

In 2011, staff in the EHS program office and in the Office of Planning Research and Evaluation (OPRE) at ACF pursued shared priorities related to strengthening EHS approaches and validating the toxic stress framework. This initiative, launched through the Buffering Children from Toxic Stress (BTS) grant program (OPRE, n.d.), aimed to investigate how stress and adversity, particularly in the context of supportive caregiving, disrupt young children’s development. The longstanding evidence of a socioeconomic gradient in health and developmental outcomes, along with emerging literature on the putative biological pathways underlying this phenomenon (e.g., Hertzman & Boyce, 2010) was particularly compelling for a program such as EHS, which predominantly serves families experiencing low incomes. EHS’s mission to promote sensitive and supportive caregivers aligned well with the potential buffering effects of such caregiving in the face of extreme stressors.

The BTS grant program aimed to test preventive parenting interventions with three primary objectives (Buffering Toxic Stress Consortium Principal Investigators et al., 2013):

1. Characterize the construct of toxic stress: This involved validating and refining measures of toxic stress, including using biological markers such as hair and salivary cortisol to assess risk in children and families.
2. Implement parent-focused interventions: These interventions, integrated into Early Head Start (EHS) programs, were designed to foster sensitive and supportive caregiving. The program also aimed to evaluate the conditions for successful implementation and identify barriers among families experiencing multiple risks.
3. Test the efficacy and added value: The goal was to determine how effective these interventions were in mitigating the negative effects of stress on both family and child outcomes, particularly in the context of multiple adversities.

This structured approach aimed to address both theoretical and practical aspects of toxic stress and intervention outcomes in high-risk populations. Designed as a cooperative agreement with substantial involvement from federal staff, the BTS grant program fostered collaboration among a consortium of investigators, EHS program partners, and federal leaders. Six grants were awarded, with each grant recipient developing and implementing interventions suited to their local EHS program and context.

To advance this goal, OPRE developed an adaptation of the Early Head Start University Partnership, known as the Buffering Toxic Stress Consortium (BTS Consortium). Through grant competition and award, this initiative brought together leading scholars in early adversity and early childhood social emotional development, their local EHS partners, and national leaders in early childhood policy and research at ACF. The consortium aimed to explore the promise of a toxic stress framework for informing prevention within the infrastructure of EHS. While each of the site independently designed risk measures and interventions that were suited to their local contexts, consortium members collaborated to identify shared measures across the six sites to facilitate shared cross-site learning and knowledge transfer at both local and national levels. The BTS consortium was intentionally designed as a collection of pilot studies, each exploring different approaches to buffering toxic stress in EHS families. While shared methodologies were encouraged across studies, sites retained autonomy in selecting interventions and implementation strategies. This flexibility was intentional, allowing for discovery-driven research, feasibility testing, and contextual adaptation, rather than strict experimental replication. By leveraging this approach, the consortium generated insights into the feasibility and effectiveness of diverse parenting interventions across varied family contexts, rather than direct comparisons of intervention effects. This design choice reflects the complexity of real-world implementation and informs future efforts to tailor early childhood interventions for different subgroups.

The BTS Consortium includes a set of six grantees who examined the implementation and efficacy of different promising parenting curriculum models in EHS. The cooperative agreements were awarded to New York University, University of Colorado Anschutz Medical Campus, University of Delaware, University of Denver, University of Maryland School of Social Work, and Washington University in St. Louis. Each grantee was focused on understanding a different parenting curriculum. See Buffering Toxic Stress Consortium (2013) for a complete listing of the EHS-University Partnership members (e.g., EHS grantee, investigators), community served, and parenting curriculum studies as an enhancement.

## Target Populations and Measures

The multiple risk factors families face across the BTS sites are similar to those faced by the larger EHS population,—including poverty, parental depression, and high rates of childhood adversity—which highlights the importance of developing precision approaches to effective interventions for this vulnerable group. The BTS consortium included six sites, each representing a range of racial, ethnic, and population demographics. These sites recruited families enrolled in EHS and tested promising interventions aimed at enhancing parent–child relationships and buffering children from experiencing a toxic stress response. The studies primarily focused on families with low incomes, with a notable overrepresentation of Latinx families (53% across all six sites, Phu et al., 2021), as well as American Indian Alaska Native (AIAN) families (82% for University of Colorado Anschutz), who are often underserved due to historical and ongoing marginalization. While the six BTS sites represented varied racial, ethnic, and population demographics, the studies did not examine within-group variability related to race and ethnicity. Instead, families were recruited based on their enrollment in Early Head Start and their experiences of multiple adversities, with interventions designed to enhance parent–child relationships and buffer against toxic stress.

Phu et al., (2021) provides a detailed descriptive analysis of sample characteristics across the six BTS sites. Notably, cross-site data revealed that a substantial proportion of families were living in poverty with 46–88% reporting annual incomes of $25,000 or less. Nearly one third of all children across the sites exhibited clinically elevated behavioral problems, and there was a high prevalence (51%) of children experiencing abuse or neglect in one of the sites (Wagner et al., 2022).

In one BTS site, approximately 34% of mothers reported experiencing at least one type of maltreatment during childhood, including physical, emotional, or sexual abuse, or physical or emotional neglect (Harden et al., 2021). In two other sites, participants reported a combined average of two adverse childhood experiences (ACEs), including maltreatment, exposure to domestic violence, household incarceration, household substance use, household mental illness, and parental divorce. These findings are generally consistent with national ACEs data (Felitti et al., 1998), where more than half of respondents report at least one ACE (most commonly parental divorce), and about one fourth report experiencing two or more categories of adversity. However, EHS families often face additional and compounding contextual risks, such as poverty-related stressors, which may increase severity and impact of ACEs within this population. Parental depression was also prevalent, with 19–31% of parents meeting clinical cutoffs for depression (Phu et al., 2021). These findings underscore the high-risk contexts in which EHS families navigate daily life, which can reduce program effectiveness (Raikes et al., 2013). As such, this population presents a valuable context for studying and developing precision approaches to effective interventions.

Across all six locations, various parenting interventions were implemented in both home and laboratory settings. These interventions were evaluated using a combination of observational, survey, and biological assessments, which measured family characteristics (e.g., demographic risk factors, income-to-needs ratio, household resource constraints, maternal adverse childhood experiences, caregiver mental health, parenting stress), parent–child relationship quality (e.g., sensitivity, emotional availability, dyadic mutuality), child outcomes (e.g., behavior problems, sleep quality, maltreatment), and physiological stress responses for both parents and children (e.g., salivary cortisol during acute stressors, diurnal cortisol patterns, and hair cortisol concentration). These comprehensive measures offered a robust approach to understanding both risk and protective factors that could either buffer or exacerbate the effects of adversity on child outcomes. For a full list of common measures, see Buffering Toxic Stress Consortium Principal Investigators et al., 2013.

## An Active Contrast: EHS Alone Compared to EHS Plus Parenting Enhancement

An important aspect of conducting research within the context of existing EHS infrastructure and service provision is the resulting overall contrast to be studied about EHS alone compared to EHS plus parenting enhancement (EHS +). Given the substantial evidence of EHS’s positive impact on children and families (Love et al., 2013; Sama-Miller et al., 2018), the key research question becomes whether adding a parenting enhancement provides additional buffering benefits beyond the main effects of EHS alone (e.g., an active contrast). This comparison allows for the examination of whether targeted enhancements in supportive parenting lead to improvements in child and family outcomes, while recognizing the need to tailor these intervention for different subgroups. Additionally, while it is imperative to conduct studies that establish causality (Gottfredson et al., 2015), relying solely on highly controlled research settings limits the applicability and translation of findings into real-world policy and practice (H.R. 4174, 115th Congress, 2017). Thus, to increase the public health impact of parenting interventions, it is useful to conduct studies in real-world community settings (Pinto et al., 2024), a strength of the body of work presented in this special issue. Notably, four of the six papers in this issue conducted rigorous randomized controlled trials to establish causality of effects (Hustedt et al., 2022; Harden et al., 2021; Liu et al., 2021; Wagner et al., 2022). In this context, the central question remains: What is the added value of enhancing supportive parenting, beyond the foundational effects of EHS programming, in promoting child development and well-being?

## The Parenting Enhancements Varied on Important Dimensions

The BTS Consortium studied five parenting enhancements grounded in social learning and attachment theories of development (see Table 1), emphasizing the critical relationship quality embedded in early childhood interactions with caregivers. These interventions have since been further developed, refined, and evaluated. The interventions, along with references to current manuals and training materials, include: Attachment and Bio-behavioral Catch-up (ABC; Dozier & Bernard, 2019), Filming Interactions to Nurture Development (FIND; Fisher et al., 2016), The Incredible Years for Toddlers (IYT; Webster-Stratton & Reid, 2018), Play and Learning Strategies (PALS; Landry et al., 2008), and Promoting First Relationships (PFR; Kelly et al., 2008). For University of Colorado Anschutz Medical Campus, Parent–Child Interaction Therapy (Eyberg et al., 1995), a live parent-coaching program, was implemented in an exploratory way to assess feasibility and cultural and contextual fit, building on prior work to increase cultural alignment for American Indian communities (BigFoot & Funderburk, 2011). Four of these six enhancements are reported in four of the six papers in this special issue (Harden et al., 2021; Hustedt et al., 2022; Liu et al., 2021; Wagner et al., 2022). While these interventions were similar in dose (e.g., 10–13 sessions) and were selected as promising approaches to buffer adversity as an enhancement to EHS, they varied in their emphasis on developmental themes, parenting strategies, fidelity protocols, and staffing requirements. These interventions targeted key developmental themes, such as fostering parental nurturance, improving responsiveness, promoting social-emotional and cognitive development, and enhancing secure parent–child relationships. Programs emphasized parental self-efficacy, understanding child signals, and promoting positive interactions to strengthen emotion regulation, language, and social skills. Interventions generally used home visits or group-based sessions, incorporating video feedback, coaching, in-the-moment commenting, and reflective observation. Sessions focused on practicing parenting skills, reviewing interactions, and promoting positive emotional climates to improve parent–child interactions and overall developmental outcomes. See Table 1 for an overview of parenting enhancements implemented and tested across BTS Consortium sites.

**Table 1:** Overview of parenting curriculum enhancements implemented across BTS Consortium sites

Parenting Curriculum Chosen as Enhancement	Developmental Themes of Focus	Intervention Approach for Strengthening Parenting
Attachment, Bio-Behavioral Catchup (ABC) – Dozier & Bernard, 2019	• Importance of parental nurturance • Following the child's lead • Importance of non-threatening, non-frightening caregiving behavior • ‘Overriding’ one’s own history and/or non-nurturing instincts	• Ten weekly home visiting sessions (parent and infant/toddler together) • Video-recorded infant-parent interactions • In-the-moment commenting • Discussion of the concepts and practical challenges • Homework assignments
Filming Interactions to Nurture Development (FIND) – Fisher et al., 2016	For caregivers exposed to childhood adversity, • Importance of focusing on the observed strengths of a child and his/her caregiver as a means of improving caregiver’s parenting self-efficacy • Assertion that interventions that attempt to correct ineffective parenting might further diminish confidence in parenting and exacerbate existing mental health problems	• Video feedback intervention program based on microsocial interaction research (parent and infant/toddler together) • Ten weekly home visit coaching sessions, with every two sessions targeting one core element of serve and return interactions • The five core elements of serve and return interactions are: (a) Sharing the child’s focus; (b) Supporting and encouraging; (c) Naming; (d) Back and forth interaction; (e) Endings and Beginnings
Incredible Years for Toddlers (IYT) – Webster-Stratton & Reid, 2018	• Importance of influence of positive relationships between parent and toddler on the progression of following benefits for the toddler: o Increased language skills and emotional regulation o Increased social skills o Increased trust and security o Decreased behavior problems	• Thirteen bi-weekly group-based sessions (parents only) • Activities to build parenting skills follow the below progression - Child-directed play - Promoting toddler language and child directed coaching - Social and emotion coaching - Praise and encouragement - Spontaneous incentives for toddlers - Handling separations and reunions - Predictable routines - Positive discipline – effective limit setting and handling misbehavior
Playing and Learning Strategies (PALS) – Landry et al., 2008	• Importance of parent empowerment and parental understanding of how their actions impact their child’s development • Importance of bonding between parent and child for child’s social-emotional, cognitive, and language skills	• Eleven weekly home visits (parent and infant/toddler together) • Coaches follow a carefully scripted session to lead parents in practicing skills and reviewing videos of their own behaviors • The parent skills of focus are: - Recognize and respond appropriately to their child’s signals - Maintain their child’s focus of attention - Stimulate language development and thinking skills - Support their child’s autonomy-seeking - Respond to limit-testing behaviors
Promoting First Relationships (PFR) – Kelly et al., 2008	• Importance of understanding factors that promote positive parent-infant relationship • Importance of using reflective observation, verbal feedback and reflective questioning • Importance of discussing social and emotional needs specific to the infant–toddler period	• Ten weekly sessions (parent and infant/toddler together) • Manualized use of specific consultation strategies such as establishing a positive emotional climate for the parent; promoting parents’ capacities to be reflective when observing their child • Service providers record short video segments of parents interacting with child and then watch them together, reviewing PFR principles, highlighting strengths, and offering suggestions

## The Six Papers in this Special Issue

The papers in this special issue highlight the importance of understanding and addressing risk and resilience associated with parenting and child outcomes and the needs of EHS families facing elevated adversity. Across several studies conducted, researchers explored the associations between family characteristics—such as economic hardship and financial strain, caregiver mental health, caregiver adverse childhood experiences, intimate partner violence, and child temperament—and outcomes related to parent–child relationship quality, parenting stress, parenting skills, children’s behavioral and biomarkers of stress regulation and behavioral problems, as well as intervention effectiveness (See Table 2 for a summary of papers).

**Table 2:** Summary of papers published in the special issue

Title	Characterizing Family Contextual Factors and Relationships with Child Behavior and Sleep Across the Buffering Toxic Stress Consortium	Emotional availability as a moderator of stress for young children and parents in two diverse Early Head Start samples	Child Temperament as a Moderator of Promoting First Relationships Intervention Effects Among Families in Early Head Start
Authors	Phu et al., 2021	Senehi et al., 2021	Hustedt et al., 2022
Data Site	All 6 Buffering Toxic Stress Consortium sites	University of Denver & University of Colorado Anschutz	University of Delaware
Parenting Curriculum	N/A—Did not test intervention, explored child, family, and contextual characteristics for BTS sample across all sites	N/A, utilized pre-intervention baseline data to assess parental emotional availability in the parent–child relationship	**Promoting First Relationships (PFR) –** Kelly et al., 2008
Study Question/Aim	(1) provide descriptive information on child and family characteristics, especially complex risk exposure and caregiver mental health, across the BTS consortium sites and (2) characterize how family characteristics relate to behavioral and sleep outcomes in children	Does maternal emotional availability buffer the association between maternal ACEs and children's HCC?	Does child temperament influence the effectiveness of PFR in (1) improving family functioning, (2) reducing parenting stress, and (3) promoting positive parent–child interactions?
Method	Descriptive statistics used to describe child and family characteristics, multi-level regression models (children nested within sites) used to test associations between family characteristics, child temperament, with children’s behavior and sleep outcomes	Moderation analyses of observational coding of maternal emotional availability using parent–child interaction videos, collected children’s HCCs, maternal report of ACEs	RCT with two groups (1) PFR with EHS and (2) waitlist-control group with EHS only
Findings	Higher family demographic risk and parental mental health problems were associated with increased behavior problems in toddlers and lower social-emotional competence in infants, poor sleep quality was associated with child negative affect and caregiver mental health problems	Higher family demographic risk and parental mental health problems were associated with increased behavior problems in toddlers and lower social-emotional competence in infants. Poor sleep quality was associated with child negative affect and caregiver mental health problems	Main Effect – Null; Moderated Effects—Child temperament moderated PFR effects on maternal parenting stress and sensitivity, PFR was especially beneficial for EHS families with children who showed high levels of surgency and negative affect
Title	Improving Caregiver Self‑Efficacy and Children’s Behavioral Outcomes via a Brief Strength‑Based Video Coaching Intervention: Results from a Randomized Controlled Trial	Maternal Psychological Risk Moderates the Impacts of Attachment-Based Intervention on Mother-Toddler Mutuality and Toddler Behavior Problems: A Randomized Controlled Trial	Parameterizing Toxic Stress in Early Childhood: Maternal Depression, Maltreatment, and HPA-Axis Variation in a Pilot Intervention Study
Authors	Liu et al., 2021	Harden et al., 2021	Wagner et al., 2022
Data Site	University of Denver	University of Maryland	Washington University
Parenting Curriculum	**Filming Interactions to Nurture Development (FIND) –** Fisher et al., 2016	**Attachment, Bio-Behavioral Catchup (ABC) –** Dozier & Bernard, 2019	**The Incredible Years for Toddlers (IYT) –** Webster-Stratton & Reid, 2018
Study Question/Aim	Tested (1) effectiveness of FIND on caregivers’ self-efficacy and (2) reduction in children internalizing and externalizing problems; (3) If caregivers’ adverse childhood experiences (ACEs) moderated FIND-related changes in parenting self-efficacy, (4) if improvement in caregivers’ self-efficacy would underlie FIND-related decreases in children’s internalizing and externalizing problems	Testing effectiveness of ABC on (1) mother-toddler dyadic mutuality and (b) to test the moderating role of maternal psychological risk (i.e., maternal maltreatment history, intimate partner violence, and mental health problems) in intervention impacts on dyadic mutuality and toddler behavior problems	(1) to validate HCC as a biomarker of HPA-axis variation; (2) to explore the associations between toxic stressors with one another and with behavioral outcomes of children in poverty; and (3) to pilot IYT
Method	Random assignment into FIND or active control	A total of 208 parent–child dyads participated in this randomized trial of EHS plus ABC	Two-stage RCT, first stage hair cortisol was validated as an index of HPA-axis variation, second stage randomized into IYT or control
Findings	FIND group improved in self-report parenting competence and self-efficacy, direct effects on children’s behavior problems not observed, FIND was more beneficial for caregivers with higher ACEs, caregivers’ improved self-efficacy in teaching tasks was linked to children’s reduced internalizing and externalizing	ABC dyads demonstrated higher dyadic mutuality post intervention compared to control; ABC’s impact on increased dyadic mutuality and reduced behavior problems were greater for children of mothers with high psychological risk	HCC is the most stable biomarker of HPA-axis activity relative to other biomarkers including salivary cortisol, urine cortisol, and heart rate variability. No significant main intervention effects but trends revealed lower rates of child abuse and neglect reports among IYT families compared to control

*HCC* hair cortisol concentration, a physiological marker of cumulative hypothalamic-pituitary-adrenal (HPA) axis activity; *ACEs* Adverse Childhood Experiences; *EHS* Early Head Start; *RCT* Randomized Controlled Trial

Phu et al. (2021) provide a comprehensive overview of family and child characteristics across all BTS sites (*n* = 1047) including family demographic risk (e.g., single parenting, less than high school education, unemployment, neighborhood risk), economic hardship (e.g., income-to-needs ratio), household resource constraints, perception of economic hardship and pressure, caregiver mental health (e.g., depression, anxiety), parent–child dysfunctional interaction, and child temperament. Additionally, using multilevel analyses they test associations between family characteristics and children’s sleep quality and behavior problems across all study participants. Findings from this study revealed greater child internalizing and externalizing symptoms in toddlers and decreased social-emotional competence in infants were associated with greater family demographic risk and parental mental health problems including depression and anxiety. Additionally, children’s lower sleep quality was associated with parental mental health problems and difficult temperament. The findings suggest that addressing caregiver mental health and financial strain could improve the effectiveness of parent–child interventions in reducing behavior and sleep issues in early childhood.

Senehi et al. (2021) explore how maternal emotional availability moderates the relationship between maternal adverse childhood experiences (ACEs) and toddlers’ physiological stress, measured by hair cortisol concentrations (HCCs) as a marker of hypothalamic–pituitary–adrenal (HPA) axis activity. The study found that higher parental ACEs were linked to elevated HCCs in children, with more pronounced increases in families where parents had six or more ACEs. However, greater emotional availability in parents moderated this relationship, demonstrating a buffering effect against the impact of parental childhood adversity on children’s stress response. In contrast, children of parents with lower emotional availability were not protected from the effects of parental ACEs, as shown by elevated HCCs. The findings also suggest that the buffering effect of higher emotional availability was particularly beneficial for children of parents with six or more ACEs compared to those with fewer ACEs. This highlights the potential of enhancing emotional availability in early life, especially for parents with greater than six ACEs, as a means of reducing toxic stress and promoting more tolerable stress responses in children, as indicated by lower HCC levels.

Hustedt et al. (2022) used a randomized controlled trial to test the effectiveness of the PFR intervention in reducing parenting stress and promoting positive parent–child interaction. Although they did not find main effects of PFR, moderated effects were observed. Specifically, child temperament moderated PFR's effects on maternal parenting stress and sensitivity. PFR was particularly beneficial for families with children displaying high levels of surgency and negative affect. Reductions in parenting stress were observed when children demonstrated higher levels of surgency, while increased parental sensitivity was observed when children exhibited higher levels of negative affect. Thus, PFR appeared to be especially valuable for EHS families with children characterized by challenging temperaments.

Liu et al. (2021) also conducted a randomized controlled trial to evaluate the effectiveness of FIND on improving caregiver self-efficacy and reducing children’s internalizing and externalizing behaviors. Results showed that caregivers in the FIND group experienced increased parenting competence and self-efficacy. Although FIND did not show direct effects on reducing children’s behavioral problems, it was especially beneficial for caregivers with greater exposure to ACEs. Improved caregiver self-efficacy in teaching tasks was linked to reductions in children’s internalizing and externalizing problems, indicating the potential of FIND to indirectly benefit children through enhancing parenting self-efficacy.

Building on earlier findings that ABC improved parenting sensitivity and positive regard (Berlin et al., 2018), infants’ use of mother-oriented emotion regulation strategies (Hepworth et al., 2020), and infant cortisol regulation during standardized stress tasks through increased maternal sensitivity (Berlin et al., 2019), Harden et al. (2021) conducted a randomized controlled trial to further evaluate the effectiveness of the ABC intervention (Dozier & Bernard, 2019). The study aimed to assess ABC's impact on mother-toddler dyadic mutuality and toddler behavior problems, particularly in the context of maternal psychological risk. While main effects suggested that mothers in the ABC group showed improvements in dyadic mutuality, moderation effects suggested that improvements in dyadic mutuality and reductions in toddlers’ behavior problems were primarily observed in families with high maternal psychological risk, such as maternal maltreatment history, intimate partner violence, and mental health problems including depression and anxiety. These findings emphasize the importance of tailoring interventions to meet the specific needs of subgroups facing significant psychological challenges.

Finally, Wagner et al. (2022) utilized various biomarkers of HPA-axis activity to validate the use of hair cortisol concentration (HCC) as a temporally stable biomarker for toxic stress response in Early Head Start (EHS) children experiencing high poverty and a high prevalence of child abuse and neglect. They found relatively low associations between maternal depression, child abuse and neglect reports, caregiver emotional availability, children’s HCC, and behavior problems, as well as trends suggesting lower reports of child abuse and neglect for parents in the IYT program. Overall, their study indicated that measuring a single risk indicator is not sufficient as a proxy for others, emphasizing the importance of comprehensively assessing both risk and compensatory influences to rigorously measure the toxic stress construct.

## Advancing Precision Approaches in Preventive Parenting Enhancements

Collectively, the six papers presented in this special issue validate different aspects of the toxic stress framework and provide insights to inform research and evaluation efforts aimed at using a precision approach to improve the effectiveness and scalability of preventive parenting enhancements for meaningful subgroups. This is a critical need in the field to enhance intervention uptake and achieve sustained, improved outcomes (Supplee & Duggan, 2019). The findings offer a comprehensive characterization of family risk and resilience profiles, highlighting multiple co-occurring and heightened levels of adversity experienced by EHS families. While traditional demographic factors—such as maternal education, family income, and child biological sex—have historically been used to characterize family risk, findings across these studies suggest that more proximal influences, such as caregiver mental health and maternal experiences of adversity, play a more critical role in intervention effectiveness. This underscores the need to move beyond broad demographic indicators and instead assess variation of experience to better understand which families benefit most from specific interventions.

Furthermore, the studies provide insights into the validity of measurement tools used to assess toxic stress and its associated characteristics and outcomes, while emphasizing the need for comprehensive, contextually tailored measurement to help identify meaningful subgroups. Comprehensive risk assessment is crucial for precision home visiting approaches, which aim to customize interventions (what works) based on specific risk factors (for whom). All six studies comprehensively characterize family and child characteristics, demonstrating the complexity of challenges these families face. For instance, Phu et al. (2021) provide an extensive overview of family and child characteristics across all sites, highlighting the link between caregiver mental health, child negative affect, and child outcomes related to behavior and sleep quality. Several studies incorporated biomarkers of stress, such as salivary and hair cortisol concentration (Senehi et al., 2021; Wagner et al., 2022), alongside traditional stress indicators, including economic hardship (Phu et al., 2021) and family and maternal psychological functioning (e.g., depression, anxiety, ACEs, maltreatment history, and intimate partner violence. Wagner et al., (2022) cautions against relying on single indicators of stress, highlighting the need for more comprehensive measurement approaches that capture both risk and protective factors in early childhood prevention programming.

Additionally, the findings presented in this special issue provide valuable insights into intervention impacts, particularly in understanding the conditions under which interventions are most effective for different families. While all projects originally expected direct effects, findings suggest that moderated effects were more predominant, reinforcing the importance of precision-based approaches and tailoring interventions to family-specific risk and resilience factors. For instance, Senehi et al., (2021) found that maternal emotional availability buffered the physiological stress response in toddlers with greater parental exposure to ACEs. Liu et al., (2021) demonstrated that the FIND intervention improved parenting self-efficacy during teaching tasks, which was related to reductions in behavior problems, while Hustedt et al. (2022) fount that PFR reduced parenting stress and enhanced parental sensitivity. Additionally, building on previously published findings from a BTS grantee that established ABC’s effects on improved maternal sensitivity (Berlin et al., 2018) on improved salivary cortisol regulation in their infants facing acute stressors (Berlin et al., 2019), Harden et al. (2021) found that the ABC intervention increased mother–child dyadic mutuality and reduced toddler behavioral problems, particularly for families with high maternal psychological risk (e.g., maternal maltreatment history, intimate partner violence, and mental health challenges).

To improve precision, it is critical to consider which specific active ingredients drive the effectiveness of preventive parenting enhancements or the “what works” (Supplee & Duggan, 2019). Across BTS interventions, parental sensitivity in ABC (Berlin et al., 2019) and self-efficacy in FIND (Liu et al., 2021) were identified as key drivers of positive impacts. However, an even greater contribution of these findings is in identifying “for whom” these interventions are most effective. Multiple studies—including those evaluating PFR (Hustedt et al., 2022), FIND (Liu et al., 2021), and ABC (Harden et al., 2021)—demonstrate that intervention effectiveness varies significantly based on family and child characteristics, such as child temperament (Hustedt et al., 2022), caregiver ACEs (Liu et al., 2021; Senehi et al., 2021), and maternal psychological risk (Harden et al., 2021).

A consistent finding across these studies is the moderating role of maternal and child characteristics in program effectiveness. Hustedt et al. (2022) found that PFR was particularly beneficial for children with high levels of surgency and negative affect, contributing to reduced parenting stress and enhanced parental sensitivity. Although moderation was not explicitly tested, similarly, Phu et al. (2021) found that greater child negative affect was associated with poorer sleep quality, underscoring the importance of child emotional characteristics in family outcomes. Maternal psychopathology and ACEs were also consistent moderators across studies. For example, Harden et al. (2021) found that the ABC intervention was most effective in enhancing mother-toddler dyadic mutuality and reducing toddler behavior problems for families with high maternal psychological risk, including maternal maltreatment history, intimate partner violence, and mental health problems including depression and anxiety.

The role of maternal ACEs—including physical and emotional abuse, neglect, sexual abuse, and household dysfunction—was particularly evident across the studies. Greater exposure to ACEs was associated with lower emotional availability (Senehi et al., 2021), lower parenting self-efficacy (Liu et al., 2021), higher child behavior problems (Liu et al., 2021) and higher child hair cortisol concentrations (Senehi et al., 2021). Additionally, Senehi et al. (2021) demonstrated that maternal emotional availability had a stronger buffering effect for children of parents with greater ACEs, whereas lower emotional availability placed children of mothers with higher ACEs at greater risk for toxic stress responses (as indicated by greater hair cortisol concentrations). Liu et al. (2021) similarly found that the FIND intervention, which aimed to improve caregiver self-efficacy, was particularly effective for caregivers with greater exposure to ACEs, indirectly benefiting child outcomes.

## Key Takeaways for Precision Approaches

The synthesis of findings across these six studies underscores the need to move beyond traditional demographic characteristics, such as child biological sex and family income, toward assessing more proximal influences such as caregiver history of adversity, mental health, sensitivity, self-efficacy, and emotional availability. This shift highlights the importance of refining measurement approaches to better capture the experiences of families, allowing for more precise and contextually relevant insights into child development and family variation. Collectively, these findings suggest that utilizing the toxic stress framework to develop tailored approaches—accounting for individual family and child risk factors—can enhance the impact of preventive parenting interventions by promoting improved parent–child relationship quality and parenting skills, reducing parenting stress, and improving child sleep, stress regulation, and behavioral outcomes. This approach is particularly effective for EHS families experiencing elevated stress or psychological risk, emphasizing the need for interventions designed to meet the specific needs of meaningfully identified subgroups to maximize their effectiveness and mitigate the adverse impacts of stress.

Additionally, findings from these studies provide strong evidence for the importance of moderated intervention effects. Rather than broad direct effects across entire samples, the results suggest that tailored approaches are essential to enhance effectiveness for specific subgroups (for whom). These results underscore that program effectiveness is not uniform across families but rather depends on key moderators, such as caregiver ACEs, psychological risk, and child temperament. Accurately identifying and assessing family-specific risk factors ensures that resources are allocated efficiently and that interventions are appropriately matched to family needs. These findings align with research advocating for a precision home-visiting approaches, where interventions are customized based on the unique needs and characteristics of families to maximize impact and efficiency (Haroz et al., 2019). Tailoring home-visiting programs to address specific risk factors, such as maternal adverse childhood experiences and psychopathology, could significantly enhance the effectiveness of these interventions (Haroz et al., 2019). Together, these findings emphasize the need for precision-based early interventions, comprehensive measurement strategies, and a focus on the protective role of positive early relationships in mitigating toxic stress responses. This is reflected in physiological stress markers and improved child behavioral outcomes, reinforcing the importance of individualized, targeted approaches in preventive parenting enhancements.

## The Role of EHS in Disrupting the Negative Impacts of Adversity: Current and Future Directions

By synthesizing these findings, this overview also seeks to provide insights into how recent OHS regulations, policies, and practices could be adapted or informed to better address the needs of EHS populations, promoting long-term resilience in families facing early adversity. EHS programming plays a pivotal role in mitigating the cascading and intergenerational impacts of adversity on young children and their families through two-generation comprehensive evidence-based supports and services designed to improve child and family well-being. This role is especially demonstrated by the evidence presented across the papers in this special issue.

The BTS consortium parenting enhancements including FIND, PFR, and ABC, were implemented across diverse EHS family contexts to improve parent–child relationship quality and strengthen supportive caregiving behaviors. Importantly, all of these interventions were delivered within the foundation of existing EHS services and supports, which already provide a comprehensive array of resources to families. This highlights the role of EHS as a platform for delivering and enhancing targeted parenting interventions, ensuring that families receive both general and specialized supports based on their needs. The findings across the special issue showcase how EHS can be leveraged to buffer children from experiencing a toxic stress response, thus promoting resilience and helping break the intergenerational cycle of adversity for families experiencing poverty, mental health problems, financial strain and economic hardship, and early experiences of adversity.

These findings also reinforce the significance of federal regulations for EHS programming, such as the Head Start Program Performance Standards (Office of Head Start, 2024a), which elevate the role of mental health supports and services for adults in the child’s life including parents as well as staff. Related to the Office of Head Start (2024a), updated policy guidance also calls for EHS and Head Start programs to develop a comprehensive, integrated early childhood mental health approach that has a key focus on promoting family well-being by providing mental health and education support services to parents, including providing training for parents to learn about how to develop safe, stable, and nurturing relationships and environments at home (Office of Head Start, 2024b). Furthermore, the expansion of EHS slots, as noted by the National Head Start Association (2022), underscores the need for more opportunities for families to receive effective early interventions.

## Key Implications and Directions for EHS Programming

- These findings emphasize the role of adult psychological risk, including depression, anxiety, history of maltreatment and ACEs (Harden et al., 2021; Liu et al., 2021; Senehi et al., 2021), as well as child temperament (Hustedt et al., 2022) as moderators of intervention effectiveness. This underscores the importance of addressing maternal mental health within parenting programs to enhance overall family wellbeing. Correspondingly, EHS programming highlights the need for an Infant Mental Health Coordinator, focused on not just the infants but also on supporting adult mental health.
- Early interventions, particularly those focusing on enhancing parenting practices and emotionally supportive caregiver-child interactions (Senehi et al., 2021), were found to be crucial in mitigating risks and promoting positive outcomes in children. Programs including FIND (Liu et al., 2021) and ABC (Harden et al., 2021) were particularly noted for their effectiveness in improving caregiver responsiveness, skills, and child behavior.
- Comprehensive risk assessment is crucial for the precision home visiting approach, which aims to customize interventions (what works) moderated based on specific risk and protective factors (for whom). Findings from BTS suggest that more resource-intensive, targeted interventions may not be necessary for all families but should be prioritized for those experiencing higher levels of adversity, psychological distress, or complex caregiving challenges. This approach promotes the efficient allocation of resources within EHS by ensuring that supplemental interventions are directed where they are most needed. As highlighted in multiple studies (Harden et al., 2021; Hustedt et al., 2022; Senehi et al., 2021), this precision-based strategy supports the development of culturally and contextually responsive programs that are most effective for specific subgroups, such as families facing high demographic stress, early childhood adversity, and maternal mental health concerns.
- The ability to accurately identify and assess these risks ensures that resources are allocated efficiently and that interventions are appropriately matched to family needs. Grounded in social determinants of health, EHS is well-positioned to focus on family demographic risk and caregiver mental health problems, prevalent risks characterizing EHS families, and to identify areas of concern that have not been a central focus, such as children’s sleep quality (Phu et al., 2021).
- Current HS and EHS regulations emphasize that all staff members play a role in supporting mental and behavioral health, underscoring the importance of a multi-disciplinary approach. This aligns with findings from BTS that highlight the moderating role of maternal mental and behavioral health and the need for coordinated efforts, including community-based participatory research and research practice partnerships, in buffering the impacts of adversity.
- Given the increased focus and prioritization of adult mental health in EHS and HS programs, it is important to consider what resources could be brought to bear for programmatic add-ins, such as ABC or FIND. By embedding these evidence-based practices into the comprehensive services of EHS and HS, these interventions can further enhance resilience among families.

## Future Directions for Parenting Enhancements to Buffer Adversity

Continued efforts to identify the most effective components of interventions and to determine for whom they work best is important. Comprehensive risk assessments, aligned with precision home visiting approaches, will allow for more tailored interventions that address specific risk factors, such as maternal history of adversity, mental health problems, current demographic stress, and child temperament. Additionally, integrating efforts to promote family economic well-being, through dual-generation or multi-pronged approaches, will further enhance the capacity of EHS to disrupt adversity’s impacts by strengthening parenting behaviors that buffer children from experiencing prolonged yet unbuffered toxic stress responses that puts them at risk for poor mental and physical health outcomes. Despite the potential benefits, these studies also highlight challenges including high cost and complexity of implementing comprehensive parenting interventions. Findings suggest that not all families may require intensive, resource-heavy interventions, and that targeted approaches based on risk assessment may enhance cost-effectiveness. Future research should explore how to leverage existing EHS services to identify families who would benefit most from supplemental interventions, ensuring efficient use of resources while maximizing impact. Additionally, future research might explore how EHS programs can leverage family and community strengths alongside addressing risk factors. Many families facing adversity also draw on cultural values, strong social networks, and adaptive parenting strategies that support resilience (Gennetian et al., 2021). Integrating these strengths into intervention models could enhance their effectiveness and sustainability. Moreover, while these studies provide valuable insights into moderated effects and subgroup-specific findings, they were not designed to directly compare interventions across contexts; thus, future research should explore how different parenting interventions perform across diverse settings to better understand their generalizability and the conditions under which they are most effective. Important next steps include asking the following question: What will it take to create cost-effective, accessible, and scalable solutions to ensure that all families can benefit?

These evolving strategies, in line with the priorities of recent policy initiatives (OHS 2024a, b) will ensure that EHS continues to be at the forefront of efforts to integrate mental and behavioral health into federal research and programming. Precision intervention models and the ongoing improvement in translating developmental science into programming and policy will further strengthen EHS’s ability to buffer toxic stress and support the mental and physical wellbeing of children and families living with adversity.

## Data Availability

This manuscript does not report original data. All data referenced are from sources cited within the manuscript.

## Footnotes

1 Latinx—In the BTS papers, the term Latinx is used to refer to participants who identified as Hispanic, Latina, or Latino in terms of parental ethnicity. We chose this term but recognize that terminology preferences vary across individuals and communities.

2 Grant recipients refer to Early Head Start (EHS) programs that receive funding from the Office of Head Start (OHS).

## Declarations

All authors are affiliated with the Administration for Children and Families, US Department of Health and Human Services, Washington, DC, United States. Neda Senehi PhD was a Society for Research in Child Development Federal Executive Branch Science and Technology Policy Fellow in the Office of Planning Research and Evaluation (OPRE) within ACF from 2021 to 2023; Aleta Meyer PhD and Alysia Blandon PhD are currently in OPRE within ACF; Mary Bruce Webb PhD is retired from OPRE; Sangeeta Parikshak PhD is currently in the Office of Head Start within ACF.

## References

[CR1] Baxter, C., Aikens, N., Tarullo, C., Ayoub, C., Roberts, J., Mondi-Rago, C., & Gaither, M. I. (2022). *Recruitment, Selection, Enrollment, and Retention Strategies with Head Start-Eligible Families Experiencing Adversity: A Review of the Literature.* OPRE Report #2022–97. Washington, DC: Office of Planning, Research, and Evaluation, Administration for Children and Families, U.S. Department of Health and Human Services.

[CR2] Berlin, L. J., Martoccio, T. L., & Jones Harden, B. (2018). Improving early head start’s impacts on parenting through attachment-based intervention: A randomized controlled trial. *Developmental Psychology,**54*(12), 2316–2327. 10.1037/dev0000592 30335427 10.1037/dev0000592

[CR3] Berlin, L. J., Martoccio, T. L., Bryce, C. I., & Harden, B. J. (2019). Improving infants’ stress-induced cortisol regulation through attachment-based intervention: A randomized controlled trial. *Psychoneuroendocrinology,**103*, 225–232. 10.1016/j.psyneuen.2019.01.005 30716550 10.1016/j.psyneuen.2019.01.005

[CR4] BigFoot, D. S., & Funderburk, B. W. (2011). Honoring children, making relatives: The cultural translation of parent-child interaction therapy for American Indian and Alaska Native families. *Journal of Psychoactive Drugs,**43*, 309–318. 10.1080/02791072.2011.628924 22400462 10.1080/02791072.2011.628924

[CR5] Blair, C., & Raver, C. C. (2012). Child development in the context of adversity: Experiential canalization of brain and behavior. *American Psychologist,**67*(4), 309–318. 10.1037/a0027493 22390355 10.1037/a0027493 PMC5264526

[CR6] Bornstein, M. H., Cluver, L., Deater-Deckard, K., Hill, N. E., Jager, J., Krutikova, S., …, Yoshikawa, H. (2022). The future of parenting programs: I. Design. *Parenting*, *22*(3), 201–234. 10.1080/15295192.2022.2087040

[CR7] Brophy-Herb, H. E., Choi, H. H., Senehi, N., Martoccio, T. L., Bocknek, E. L., Babinski, M., Krafchak, S., Accorsi, C., Azmoudeh, R., & Schiffman, R. (2022). Stressed mothers receiving infant mental health-based Early Head Start increase in mind-mindedness. *Frontiers in Psychology,**13*, 897881. 10.3389/fpsyg.2022.897881 35719560 10.3389/fpsyg.2022.897881 PMC9201035

[CR8] Buffering Toxic Stress Consortium Principal Investigators, Meyer, A., & Fortunato, C. K. (2013). Parenting interventions in Early Head Start: The Buffering Toxic Stress consortium. *Zero to Three,**34*, 73–86.

[CR9] Chen, M., & Chan, K. L. (2016). Effects of parenting programs on child maltreatment prevention: A meta-analysis. *Trauma, Violence, & Abuse,**17*(1), 88–104. 10.1177/1524838014566718 10.1177/1524838014566718 25573846

[CR10] Dozier, M., & Bernard, K. (2019). *Coaching parents of vulnerable infants: The Attachment and Biobehavioral Catch-up approach*. Guilford Press.

[CR11] Evans, G. W., & Schamberg, M. A. (2009). Childhood poverty, chronic stress, and adult working memory. *Proceedings of the National Academy of Sciences,**106*(16), 6545–6549. 10.1073/pnas.0811910106 10.1073/pnas.0811910106 PMC2662958 19332779

[CR12] Eyberg, S. M., Boggs, S. R., & Algina, J. (1995). Parent-child interaction therapy: A psychosocial model for the treatment of young children with conduct problem behavior and their families. *Psychopharmacology Bulletin,**31*, 83–91. 7675994

[CR13] Fagan, A. A., Bumbarger, B. K., Barth, R. P., Bradshaw, C. P., Cooper, B. R., Supplee, L. H., & Walker, D. K. (2019). Scaling up evidence-based interventions in us public systems to prevent behavioral health problems: Challenges and opportunities. *Prevention Science,**20*(8), 1147–1168. 10.1007/s11121-019-01048-8 31444621 10.1007/s11121-019-01048-8 PMC6881430

[CR14] Felitti, V. J., Anda, R. F., Nordenberg, D., Williamson, D. F., Spitz, A. M., Edwards, V., & Marks, J. S. (1998). Relationship of childhood abuse and household dysfunction to many of the leading causes of death in adults: The Adverse Childhood Experiences (ACE) Study. *American Journal of Preventive Medicine,**14*(4), 245–258. 10.1016/S0749-3797(98)00017-8 9635069 10.1016/s0749-3797(98)00017-8

[CR15] Fisher, P. A., Frenkel, T. I., Noll, L. K., Berry, M., & Yockelson, M. (2016). Promoting healthy child development via a two-generation translational neuroscience framework: The Filming Interactions to Nurture Development video coaching program. *Child Development Perspectives,**10*(4), 251–256. 10.1111/cdep.12195 28936231 10.1111/cdep.12195 PMC5603284

[CR16] Forry, N. D., Moodie, S., Simkin, S., & Rothenberg, L. (2011). Family-provider relationships: A multidisciplinary review of high-quality practices and associations with family, child, and provider outcomes (Issue Brief OPRE 2011–26a). Office of Planning, Research and Evaluation, Administration for Children and Families, U.S. Department of Health and Human Services. Retrieved August 3, 2024, from https://www.acf.hhs.gov/sites/default/files/documents/opre/family_provider_multi.pdf

[CR17] Gennetian, L. A., Cabrera, N., Crosby, D., Guzman, L., Smith, J. M., & Wildsmith, E. (2021). A strength-based framework for realizing Latino young children’s potential. *Policy Insights from the Behavioral and Brain Sciences,**8*(2), 152–159. 10.1177/23727322211033618

[CR18] Gottfredson, D. C., Cook, T. D., Gardner, F. E. M., Gorman-Smith, D., Howe, G. W., Sandler, I. N., & Zafft, K. M. (2015). Standards of evidence for efficacy, effectiveness, and scale-up research in prevention science: Next generation. *Prevention Science,**16*(7), 893–926. 10.1007/s11121-015-0555-x 25846268 10.1007/s11121-015-0555-x PMC4579256

[CR19] Gunnar, M. R., Hostinar, C. E., Sanchez, M. M., Tottenham, N., & Sullivan, R. M. (2015). Parental buffering of fear and stress neurobiology: Reviewing parallels across rodent, monkey, and human models. *Social Neuroscience,**10*(5), 474–478. 10.1080/17470919.2015.1070198 26234160 10.1080/17470919.2015.1070198 PMC5198892

[CR20] H.R. 4174, 115th Cong. (2017). Foundations for Evidence-Based Policymaking Act of 2018. Retrieved August 3, 2024, from https://www.congress.gov/bill/115th-congress/house-bill/4174

[CR21] Harden, B. J., Sandstrom, H., & Chazan-Cohen, R. (2012). Early Head Start and African American families: Impacts and mechanisms of child outcomes. *Early Childhood Research Quarterly,**27*(4), 572–581. 10.1016/j.ecresq.2012.07.006

[CR22] Harden, B. J., Martoccio, T. L., & Berlin, L. J. (2021). Maternal psychological risk moderates the impacts of attachment-based intervention on mother-toddler mutuality and toddler behavior problems: A randomized controlled trial. *Prevention Science, (this issue)*. 10.1007/s11121-021-01281-0 10.1007/s11121-021-01281-0 34448111

[CR23] Haroz, E. E., Ingalls, A., Kee, C., Goklish, N., Neault, N., Begay, M., & Barlow, A. (2019). Informing precision home visiting: Identifying meaningful subgroups of families who benefit most from Family Spirit. *Prevention Science,**20*(8), 1244–1254. 10.1007/s11121-019-01039-9 31432381 10.1007/s11121-019-01039-9 PMC7082862

[CR24] Hepworth, A. D., Berlin, L. J., Martoccio, T. L., Cannon, E. N., Berger, R. H., & Jones Harden, B. (2020). Supporting infant emotion regulation through attachment-based intervention: A randomized controlled trial. *Prevention Science,**21*(5), 702–713. 10.1007/s11121-020-01127-1 32388694 10.1007/s11121-020-01127-1

[CR25] Hertzman, C., & Boyce, T. (2010). How experience gets under the skin to create gradients in developmental health. *Annual Review of Public Health,**31*(1), 329–347. 10.1146/annurev.publhealth.012809.103538 20070189 10.1146/annurev.publhealth.012809.103538

[CR26] Home Visiting Applied Research Collaborative. (n.d.). *Precision paradigm framework*. Retrieved August 3, 2024, from https://hvresearch.org/precision-paradigm-framework/

[CR27] Hostinar, C. E., Sullivan, R. M., & Gunnar, M. R. (2014). Psychobiological mechanisms underlying the social buffering of the hypothalamic–pituitary–adrenocortical axis: A review of animal models and human studies across development. *Psychological Bulletin,**140*(1), 256–282. 10.1037/a0032671 23607429 10.1037/a0032671 PMC3844011

[CR28] Hustedt, J. T., Hooper, A., Hallam, R. A., Vu, J. A., Han, M., & Ziegler, M. (2022). Child temperament as a moderator of promoting first relationships intervention effects among families in Early Head Start. *Prevention Science (this issue)*. 10.1007/s11121-022-01340-0 10.1007/s11121-022-01340-0 PMC12053191 35061166

[CR29] Kelly, J. F., Zuckerman, T., & Rosenblatt, S. (2008). Promoting first relationships: A relationship-focused early intervention approach. *Infants & Young Children,**21*(4), 285–295. 10.1097/01.IYC.0000336541.37379.0e

[CR30] Knudsen, E. I. (2004). Sensitive periods in the development of the brain and behavior. *Journal of Cognitive Neuroscience,**16*(8), 1412–1425. 10.1162/0898929042304796 15509387 10.1162/0898929042304796

[CR31] Landry, S. H., Smith, K. E., Swank, P. R., & Guttentag, C. (2008). A responsive parenting intervention: The optimal timing across early childhood for impacting maternal behaviors and child outcomes. *Developmental Psychology,**44*(5), 1335–1353. 10.1037/a0013030 18793067 10.1037/a0013030 PMC2570562

[CR32] Leijten, P., Gardner, F., Melendez-Torres, G. J., van Aar, J., Hutchings, J., Schulz, S., Knerr, W., & Overbeek, G. (2019). Meta-analyses: Key parenting program components for disruptive child behavior. *Journal of the American Academy of Child and Adolescent Psychiatry,**58*(2), 180–190. 10.1016/j.jaac.2018.07.900 30738545 10.1016/j.jaac.2018.07.900

[CR33] Liu, S., Phu, T., Dominguez, A., Hurwich-Reiss, E., McGee, D., Watamura, S., & Fisher, P. (2021). Improving caregiver self-efficacy and children’s behavioral outcomes via a brief strength-based video coaching intervention: Results from a randomized controlled trial. *Prevention Science (this Issue)*. 10.1007/s11121-021-01251-6 10.1007/s11121-021-01251-6 33961176

[CR34] Love, J., Kisker, E., Ross, C., Raikes, H., Constantine, J., Boller, K., Brooks-Gunn, J., Chazan-Cohen, R., Tarullo, L. B., Brady-Smith, C., Fuligni, A. S., Schochet, P. Z., Paulsell, D., & Vogel, C. (2005). The effectiveness of Early Head Start for 3-year-old children and their parents: Lessons for policy and programs. *Developmental Psychology,**41*, 885–901. 10.1037/0012-1649.41.6.885 16351335 10.1037/0012-1649.41.6.88

[CR35] Love, J., Kisker, E., Ross, C. M., Schochet, P. Z., Brooks-Gunn, J., Paulsell, D., et al. (2002). Making a Difference in the Lives of Infants and Toddlers and their Families: The Impacts of Early Head Start. Volumes I-III: Final Technical Report [and] Appendixes [and] Local Contributions to Understanding the Programs and Their Impacts. Washington, DC: U.S. Department of Health and Human Services, Head Start Bureau. Retrieved August 3, 2024, from https://eric.ed.gov/?id=ED472186

[CR36] Love, J. M., Chazan-Cohen, R., Raikes, H., & Brooks-Gunn, J. (Eds.) (2013). What makes a difference: Early Head Start evaluation findings in a developmental context*. Monographs of the Society for Research in Child Development*, *78*(1), 1-173.10.1111/j.1540-5834.2012.00699.x 10.1111/j.1540-5834.2012.00699.x 23425422

[CR37] Luby, J. L., Barch, D. M., Belden, A., Gaffrey, M. S., Tillman, R., Babb, C., Nishino, T., Suzuki, H., & Botteron, K. N. (2012). Maternal support in early childhood predicts larger hippocampal volumes at school age. *Proceedings of the National Academy of Sciences,**109*(8), 2854–2859. 10.1073/pnas.1118003109 10.1073/pnas.1118003109 PMC3286943 22308421

[CR38] Lupien, S. J., McEwen, B. S., Gunnar, M. R., & Heim, C. (2009). Effects of stress throughout the lifespan on the brain, behaviour, and cognition. *Nature Reviews Neuroscience,**10*(6), 434–445. 10.1038/nrn2639 19401723 10.1038/nrn2639

[CR39] Masten, A. S., spsampsps Cicchetti, D. (2016). Resilience in development: Progress and transformation. In D. Cicchetti (Ed.), *Developmental psychopathology: Risk, resilience, and intervention* (3rd ed., pp. 271–333). John Wiley spsampsps Sons, Inc. 10.1002/9781119125556.devpsy406

[CR40] Masten, A. S., & Barnes, A. J. (2018). Resilience in children: Developmental perspectives. *Children,**5*(7), 98. 10.3390/children5070098 30018217 10.3390/children5070098 PMC6069421

[CR41] McEwen, C. A., & McEwen, B. S. (2017). Social structure, adversity, toxic stress, and intergenerational poverty: An early childhood model. *Annual Review of Sociology,**43*(1), 445–472. 10.1146/annurev-soc-060116-053252

[CR42] Mingebach, T., Kamp-Becker, I., Christiansen, H., & Weber, L. (2018). Meta-meta-analysis on the effectiveness of parent-based interventions for the treatment of child externalizing behavior problems. *PLoS ONE,**13*(9), e0202855. 10.1371/journal.pone.0202855 30256794 10.1371/journal.pone.0202855 PMC6157840

[CR43] National Head Start Association. (2022). Early Head Start facts and figures: A proven support for children, pregnant women, and families. Retrieved September 3, 2024, from https://nhsa.org/wp-content/uploads/2021/12/Early-Head-Start-Facts-and-Figures_21-22-1.pdf

[CR44] National Head Start Association. (2023). *Head start workforce brief (February 2023)*. Retrieved August 3, 2024, from https://nhsa.org/wp-content/uploads/2023/03/2023.02-Workforce-Brief.pdf

[CR45] Nomaguchi, K., & Milkie, M. A. (2020). Parenthood and well-being: A decade in review. *Journal of Marriage and Family,**82*(1), 198–223. 10.1111/jomf.12646 32606480 10.1111/jomf.12646 PMC7326370

[CR46] Obradović, J., & Boyce, W. T. (2009). Individual differences in behavioral, physiological, and genetic sensitivities to contexts: Implications for development and adaptation. *Developmental Neuroscience,**31*(4), 300–308. 10.1159/000216541 19546567 10.1159/000216541

[CR47] Office of Head Start. (2024a). Head Start program performance standards. Administration for Children and Families, U.S. Department of Health and Human Services. Retrieved August 3, 2024, from https://headstart.gov/policy/45-cfr-chap-xiii

[CR48] Office of Head Start. (2024b). Promoting the integration of behavioral health in Head Start (ACF-IM-HS-20–01). Administration for Children and Families, U.S. Department of Health and Human Services. Retrieved August 3, 2024, from https://www.acf.hhs.gov/sites/default/files/documents/ecd/Head-Start-Mental-Health-IM.pdf

[CR49] Office of Head Start. (n.d.). Early Head Start programs. Head Start | ECLKC. Retrieved August 3, 2024, from https://headstart.gov/programs/article/early-head-start-programs?redirect=eclkc

[CR50] Office of Planning, Research & Evaluation. (n.d.). Early Head Start University Partnership Grants: Buffering children from toxic stress (2011). *Administration for Children and Families*. Retrieved August 3, 2024, from https://www.acf.hhs.gov/opre/project/early-head-start-university-partnership-grants-buffering-children-toxic-stress-2011

[CR51] Phu, T., Miles, E., Dominguez, A., Hustedt, J., Watamura, S. E., BTS Consortium Principal Investigators. (2021). Characterizing family contextual factors and relationships with child behavior and sleep across the buffering toxic stress consortium. *Prevention Science (this issue)*. 10.1007/s11121-021-01243-6 10.1007/s11121-021-01243-6 PMC12053054 34036462

[CR52] Pinto, R., Canário, C., Leijten, P., Rodrigo, M. J., & Cruz, O. (2024). Implementation of parenting programs in real-world community settings: A scoping review. *Clinical Child and Family Psychology Review,**27*(1), 74–90. 10.1007/s10567-023-00465-0 38062309 10.1007/s10567-023-00465-0 PMC10920434

[CR53] Raikes, H. H., Vogel, C., & Love, J. M. (2013). Family subgroups and impacts at ages 2, 3, and 5: Variability by race/ethnicity and demographic risk. What makes a difference: Early Head Start evaluation findings in a developmental context. *Monographs of the Society for Research in Child Development,**78*(1), 64–92. 10.1111/j.1540-5834.2012.00703.x 10.1111/j.1540-5834.2012.00699.x 23425422

[CR54] Raikes, H. H., Roggman, L. A., Peterson, C. A., Brooks-Gunn, J., Chazan-Cohen, R., Zhang, X., & Schiffman, R. F. (2014). Theories of change and outcomes in home-based Early Head Start programs. *Early Childhood Research Quarterly,**29*(4), 574–585. 10.1016/j.ecresq.2014.05.003

[CR55] Sama-Miller, E., Akers, L., Mraz-Esposito, A., Zukiewicz, M., Avellar, S., Paulsell, D., & Del Grosso, P. (2018). Home visiting evidence of effectiveness review: Executive summary (OPRE Report 2018–113). Office of Planning, Research and Evaluation, Administration for Children and Families, U.S. Department of Health and Human Services. Retrieved August 3, 2024 from https://acf.gov/sites/default/files/documents/opre/HomVEE_Executive%20Summary%20October%202018_0.pdf

[CR56] Sandstrom, H., Kuhns, C., & Drukker, D. (2024). *Turnover patterns among Early Head Start teachers and home visitors: A snapshot before and after the height of the COVID-19 pandemic*. Urban Institute. Retrieved August 3, 2024 from https://www.urban.org/research/publication/turnoverpatterns-among-early-head-start-teachers-and-home-visitors

[CR57] Schmit, S., & Walker, C. (2016). Disparate access: Head Start and CCDBG data by race and ethnicity. Center for Law and Social Policy (CLASP). Retrieved August 3, 2024, from https://www.clasp.org/sites/default/files/public/resources-and-publications/publication-1/Disparate-Access.pdf

[CR58] Senehi, N., Flykt, M., Biringen, Z., Laudenslager, M. L., Watamura, S. E., Garrett, B. A., Kominsky, T. K., Wurster, H. E., & Sarche, M. (2021). Emotional availability as a moderator of stress for young children and parents in two diverse Early Head Start samples. *Prevention Science (this Issue)*. 10.1007/s11121-021-01307-7 10.1007/s11121-021-01307-7 PMC12053350 34773574

[CR59] Shonkoff, J. P., & Fisher, P. A. (2013). Rethinking evidence-based practice and two-generation programs to create the future of early childhood policy. *Development and Psychopathology,**25*(4pt2), 1635–1653. 10.1017/S0954579413000813 24342860 10.1017/S0954579413000813 PMC4745587

[CR60] Shonkoff, J. P., Boyce, W. T., & McEwen, B. S. (2009). Neuroscience, molecular biology, and the childhood roots of health disparities: Building a new framework for health promotion and disease prevention. *JAMA,**301*(21), 2252–2259. 10.1001/jama.2009.754 19491187 10.1001/jama.2009.754

[CR61] Shonkoff, J. P., Garner, A. S., Committee on Psychosocial Aspects of Child and Family Health, Committee on Early Childhood, Adoption, and Dependent Care, Section on Developmental and Behavioral Pediatrics. (2012). The lifelong effects of early childhood adversity and toxic stress. *Pediatrics,**129*(1), e232–e246. 10.1542/peds.2011-2663 22201156 10.1542/peds.2011-2663

[CR62] Supplee, L. H., & Duggan, A. (2019). Innovative research methods to advance precision in home visiting for more efficient and effective programs. *Child Development Perspectives,**13*(3), 173–179. 10.1111/cdep.12334 31598130 10.1111/cdep.12334 PMC6774294

[CR63] Supplee, L. H., & Metz, A. (2015). Opportunities and challenges in evidence-based social policy. *Social Policy Report*, *28*(4), 1–16. Retrieved August 5, 2024, from https://www.srcd.org/sites/default/files/file-attachments/spr_28_4.pdf

[CR64] Supplee, L. H., Parekh, J., & Johnson, M. (2018). Principles of precision prevention science for improving recruitment and retention of participants. *Prevention Science,**19*, 689–694. 10.1007/s11121-018-0884-7 29532364 10.1007/s11121-018-0884-7

[CR65] Wagner, R. E., Jonson-Reid, M., Drake, B., Kohl, P. L., Pons, L., Zhang, Y., Fitzgerald, R. T., Laudenslager, M. L., & Constantino, J. N. (2022). Parameterizing toxic stress in early childhood: Maternal depression, maltreatment, and HPA-axis variation in a pilot intervention study. *Prevention Science,**23*(4), 636–647. 10.1007/s11121-022-01366-4 35606570 10.1007/s11121-022-01366-4 PMC12053303

[CR66] Webster-Stratton, C., & Reid, M. J. (2018). The Incredible Years parents, teachers, and children training series: A multifaceted treatment approach for young children with conduct problems. In J. R. Weisz & A. E. Kazdin (Eds.), *Evidence-based psychotherapies for children and adolescents* (3rd ed., pp. 122–141). The Guilford Press.

